# The efficacy and safety of acupoint injection for diabetic gastroparesis: A protocol for systematic review and meta-analysis

**DOI:** 10.1097/MD.0000000000023086

**Published:** 2020-11-06

**Authors:** Tao Long, Rensong Yue, Tingchao Wu, Chenyi Xu, Maoyi Yang

**Affiliations:** Hospital of Chengdu University of Traditional Chinese Medicine, Chengdu, PR China.

**Keywords:** acupoint injection, diabetic gastroparesis, protocol, systematic review

## Abstract

**Background:**

Diabetic gastroparesis (DGP) is one of the common complications of diabetes. Accumulated evidences have shown that acupoint injection is beneficial for the clinical treatment of diabetic gastroparesis. However, there is currently no systematic review to assess this therapy. This program aims to evaluate the effectiveness and safety of this therapy for the patients with DGP.

**Methods and analysis:**

Literature search will be conducted via following electronic bibliographic databases from inception to Aug 2020: the Cochrane Library, PubMed, MEDLINE, Web of Science, EMBASE, Springer, China National Knowledge Infrastructure (CNKI), China Biology Medicine (CBM), Chinese Scientific Journal Database (VIP), Wan-Fang Database. All randomized controlled trials published in English or Chinese related to acupoint injection for DGP will be included. The primary outcome is the total effective rate. The secondary outcomes are the change of motilin and gastrin levels before and after the treatment. Two researchers will be responsible for the selection of study, extraction of data, and assessment of study quality independently. RevMan V5.3 Software will be used for assessing the risk of bias and synthesizing data.

**Results:**

This study will provide a high-quality synthesis of current available evidence for the treatment of DGP with this therapy clinically.

**Conclusion:**

The conclusions of our study will provide new evidence to judge whether acupoint injection is an effective intervention for patients suffered from DGP.

**OSF registration number:**

osf.io/ms58j.

## 1 Introduction

Diabetic gastroparesis (DGP) is a common complication of diabetes characterized by delayed gastric emptying with associated upper gastrointestinal symptoms in the absence of any mechanical obstruction.^[1,2]^ The main symptoms of DGP include prolonged postprandial fullness, early satiety, nausea, vomiting, anorexia, weight loss, with or without abdominal pain.^[3–6]^ Due to delayed gastric emptying, DGP is easy to bring about impaired glycemic control, poor nutrition and hydration status, even worse frequent hospitalizations.^[1]^

As a population-based historical cohort study reported, which lasted for 10 years in Rochester US, the cumulative morbidity of diabetic gastroparesis among patients with type 1 diabetes, type 2 diabetes, and non-diabetic controls is about 5.2%, 1.0%, and 0.2% respectively.^[7]^ Besides, the people, who has significant relation with female sex, longer duration of diabetes, older age, and more frequent severe hypoglycemic episodes, is more susceptible to DGP.^[8–10]^ Another study from the UK shows that prevalence of gastroparesis greatly rose in 2016, compared to 2004. Of those with gastroparesis, 37.5% were caused by diabetes statistically, while patients with DGP had an almost 2 fold mortality to those with idiopathic gastroparesis.^[11]^ With the prevalence of diabetes increases year by year, the number of DGP keeps growing. DGP can lead to slump decline on productivity of individuals and heavy economic burdens on social healthcare resources. According to National Emergency Department Sample (NEDS), the aggregate charges for gastroparesis in the United States has increased to $5.92 million, and the bill will continue to grow exponentially.^[12]^

The routine therapies for DGP include nutritional assessment, lifestyle modifications, tight glycemic control, oral medications like prokinetics, antiemetics, and surgical treatments for refractory cases.^[1,13,14]^ Stabilize glycemic control is the basic management for DGP. Besides, nutritional assessment and lifestyle modifications are favorable but not easily followed. In terms of drugs application, prokinetic agents are beneficial to the rate of gastric emptying improvement, but not the symptoms related to DGP. As the first-line therapy, antiemetic agents are used to alleviate symptoms and regulate nutritional intake, but they have side effects. Gastric electrical stimulation can alleviate symptoms and the efficacy is persistent, while weight gain in some patients is not conducive to the control of diabetes.^[15,16]^

Acupoint injection is an alternative and complementary therapy that involves treating diseases by infusing injectable medication into patients specific acupoints. This technology is featured with low dosage and quick effect via the combination of traditional Chinese medicine (TCM) with western medicine, and is considered to possess more sustained effects than traditional acupuncture, or normal intramuscular injection^[17]^, even though the detailed mechanism of the effect is not fully understood.

TCM theory holds that stimulating acupoints is an effective method to stimulate the meridian and collateral systems, thus promoting Qi to regulate the function of the zang-fu organs, cure diseases and improve health. Zusanli (ST 36), the classic acupoint along the stomach meridian, is often used for patients with functional gastrointestinal disease (FGID) in TCM. Some studies have pointed out that stimulation at zusanli (ST 36) can significantly improve gastric motility, restore impaired gastric slow waves, and increase the amount of mucosal blood flow.^[18–20]^ Additionally, acupuncture therapy is usually used to treat diabetes and other diabetic complications, aiming at improving glucose tolerance and insulin sensitivity.^[21,22]^

Acupoint injection is widely used as a supplement therapy for patients with DGP in recent years, and the positive efficiency has attracted more and more attention from the public. However, due to the absence of critically assessed clinical evidence to evaluate therapeutic effect of acupoint injection for DGP, many patients still not benefit from this treatment. Therefore, we conducted this systematic review and meta-analysis to appraise the effectiveness and safety of acupoint injection for DGP.

## 2 Methods

### 2.1 Study registration

This protocol has been registered. OSF registration number: osf.io/ms58j. The protocol report is on the basis of the Preferred Reporting Items for Systematic Reviews and Meta-Analyses Protocols (PRISMA-P) statement guidelines.^[23]^

### 2.2 Inclusion criteria

#### 2.2.1 Types of studies

Randomized controlled trials and quasi-randomized controlled trials of acupoint injection therapy for DGP will be included, without restrictions on publication status and language, until September 20, 2020. The following types of studies, such as animal mechanism studies, case reports, self-controlled, controlled clinical trials and random crossover studies will be excluded.

#### 2.2.2 Types of participants

Participants who were 18 years or older and diagnosed as diabetes with dyspeptic symptoms will be included regardless of gender, ethnicity, education, and economic position. Patients with pylorus obstruction or gastric ulcer will be excluded by gastroscopy, ultrasound or barium X-ray.

#### 2.2.3 Types of interventions and comparisons

Interventions of the observation group will include simple acupoint injection and acupoint injection combined with other conventional treatment. Comparison interventions involve sham acupuncture, western medicine, placebo, other conventional treatment or no treatment. Any type of injected medication will be included, but acupoint injection should be the only difference between the 2 groups.

#### 2.2.4 Types of outcomes

The primary outcomes will be evaluated by gastroparesis Cardinal Symptom Index (GCSI) or similar rating scales of dyspeptic symptoms. The secondary outcomes will be gastric emptying which is evaluated by scintigraphy or radio-opaque markers. Adverse events will also be measured as secondary outcomes for safety evaluation.

### 2.3 Data sources

The following electronic databases will be searched from inception to Sep 2020: the Cochrane Library, PubMed, MEDLINE, Web of Science, EMBASE, Springer, China National Knowledge Infrastructure, China Biology Medicine, Chinese Scientific Journal Database and Wan-Fang Database. All RCTs related to acupoint injection for DGP will be included regardless of language restrictions. In addition, we also plan to scan reference lists of identified publications and meeting proceedings. Other unpublished conference articles will be manually searched.

### 2.4 Search strategy

The search strategy on PubMed is shown in Table 1. The following search terms will be used: acupoint injection (eg, “point injection”); diabetic gastroparesis (eg, “diabetic gastroparesis”); randomized controlled trial (eg, “randomized controlled trial” or “controlled clinical trial” or “random allocation” or “randomized” or “randomly” or “clinical trial”). The equivalent search terms will be used in the Chinese electronic databases.

**Table 1:** Search strategy for the PubMed database.

Number	Search items
1	Acupoint injection
2	Point injection
3	Acupuncture point injection
4	1 or 2–3
5	Diabetic gastroparesis
6	Diabetic gastric paralyze
7	Mellitus gastroparesis
8	Diabetic gastric paralyze
9	5 or 6–8
10	Randomized controlled trial
11	Randomized
12	Randomly
13	Clinical trial
14	Controlled clinical trial
15	Placebo
16	Trial
17	RCT
18	10 or 11–17
19	4 and 9 and 18

### 2.5 Data collection and analysis

#### 2.5.1 Selection of studies

**Figure 1:**
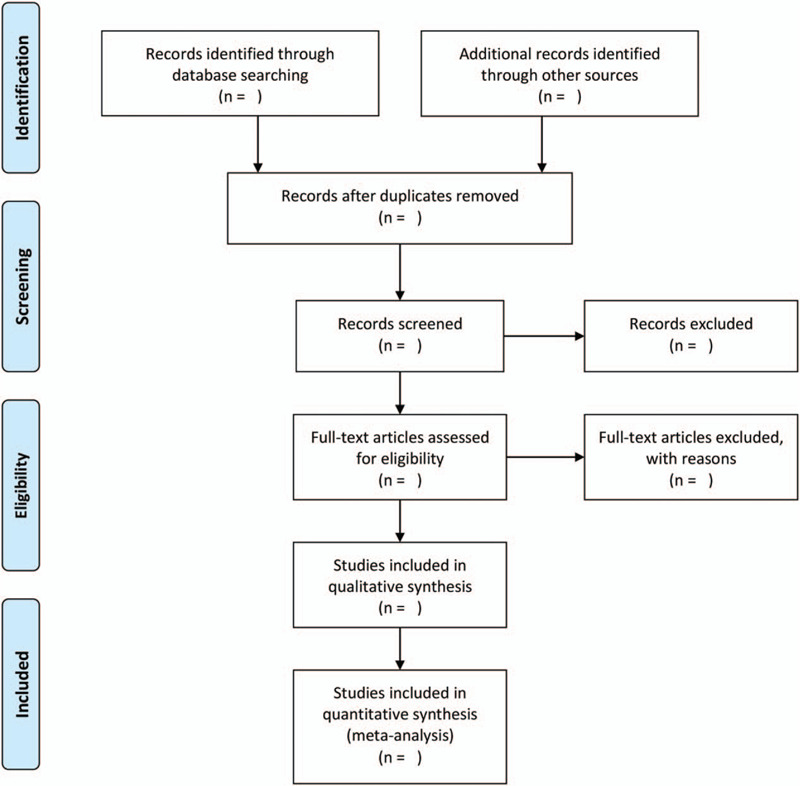
Flow chart of study selection.

To identify eligible trials and eliminate duplicate or irrelevant articles, 2 reviewers will review and screen the titles, abstracts and keywords of all retrieved literature in strict line with the inclusion and exclusion criteria independently. We will contrive to obtain the full text of all possibly eligible studies, and manage qualified articles by using EndNote software (V.X8). If there exist any disagreements, the 2 reviewers will perform a discussion and the third author will be responsible for the arbitration. The detailed process of selecting articles is shown in a PRISMA flow chart (Fig. 1).

#### 2.5.2 Data extraction and management

The data of the qualified articles will be extracted and put into a standardized, pre-set data form by 2 independent reviewers. The following basic information in each study we need to extract will include: article general information (such as year of publication, country), participant characteristics(such as age, sex), inclusion and exclusion criteria, sample size, methods, randomization, blinding methods, intervention measure of experimental and control group, outcome measures, results, follow-up, adverse reactions, and other information. If there is any disagreement in the process, the third reviewer will join in the discussion and make the final judge. When the data in articles is insufficient, we will contact the authors for further information.

#### 2.5.3 Assessment of risk of bias in included studies

Two reviewers will use the Cochrane Collaborative tool to independently evaluate the risk of bias in all included studies. We will assess the following areas of the studies: sequence generation, allocation concealment, blinding of participants and assessors, blinding of outcome assessment, incomplete outcome data, selective outcome reporting, and other sources of bias. The risk of bias will be categorized into 3 levels: low risk, high risk, and unclear. If the items are unclear or insufficient, we will contrive to contact the author for further information. Any disagreements will be resolved through the discussion with the third reviewer.

#### 2.5.4 Measures of treatment effect

Data analysis and synthesis will be performed on RevMan V.5.3. For the dichotomous data, we will use risk ratio (RR) with 95% confidence intervals (CIs) to analyze. For the continuous data with no heterogeneity, mean difference (MD) or standard mean difference (SMD) will be used to measure the treatment effect of 95% CIs. When we examine the significant heterogeneity in continuous data, a random effects model will be used.

#### 2.5.5 Unit of analysis issues

The analytical unit will be the individual patient.

#### 2.5.6 Management of missing data

In order to retrieve the missing or insufficient data of the primary results, we will contrive to contact the corresponding authors of the included articles by sending emails or making a call. If missing data is not available, we will exclude the incompetent studies from the sensitivity analysis.

#### 2.5.7 Assessment of heterogeneity

Standard Chi-Squared test will be used to detect statistical heterogeneity and *I*^2^ test will be used to quantify inconsistency. If *P* > .05 and *I*^2^ < 50%, studies will indicate homogeneity and the fixed-effects model will be applied. When *P* < .05 and *I*^2^ > 50%, we consider significant heterogeneity in the included studies, and use subgroup analysis to seek the possible cause. If significant heterogeneity is detected, the random-effects model will be applied.

#### 2.5.8 Assessment of reporting biases

Reporting biases will be detected by funnel plot if more than 10 studies are included. If the funnel plot is found to be asymmetrical, we will analyze the cause by using Egger method. When *P* < .05, the publication bias is considered to be significant.

#### 2.5.9 Data synthesis

Data analysis and quantitative data synthesis will be conducted with RevMan V.5.3. If no substantial statistical heterogeneity is detected, fixed-effect model will be applied for data synthesis. If we detect substantial statistical heterogeneity, we will use random-effects model, and explore the possible cause from a clinical and methodological perspective with providing a descriptive or subgroup analysis.

#### 2.5.10 Subgroup analysis

To explain heterogeneity, subgroup analysis will be conducted based on potential factors including different types of injected medication, acupoints, control interventions and different outcomes.

#### 2.5.11 Sensitivity analysis

In order to verify the robustness of the review conclusions, sensitivity analyses will be conducted for the primary outcomes. The impacts of following factors will be assessed, including sample size, study design, methodological quality and missing data. After low quality study is excluded, the analysis will be repeated.

#### 2.5.12 Grading the quality of evidence

The evidence quality of obtained results will be evaluated via the Grade of Recommendations Assessment, Development, and Evaluation.^[24]^ The assessment includes limitations of the study design, inconsistency, discontinuities, imprecision of results, indirectness, and publication bias. The quality of evidence will be divided into 4 levels: high, medium, low, and very low.

## 3 Ethics and dissemination

Ethical approval is not necessary because the data we adopted are not individual. The results of our study will be published at a peer-reviewed journal or presented at relevant conferences.

## 4 Discussion

Acupoint injection, which has attracted more and more attention due to the efficacy and safety in treating diabetic gastroparesis, becomes a kind of routine therapy for diabetic gastroparesis in China. However, there is no systematic and comprehensive meta-analysis on the therapeutic effect of acupoint injection for diabetic gastroparesis. Therefore, further large randomized controlled trials are needed in the future.

This systematic review will be the first to assess the effectiveness and safety of acupoint injection for diabetic gastroparesis. The review will be divided into 4 parts: identification, the inclusion of literature, data extraction, and data synthesis. We believe that this review will offer practitioners more convincing evidence in decision-making for considering acupoint injection as an alternative therapy for DGP, and will provide more options for patients to cure disease.

## Author contributions

**Conceptualization:** Tao Long, Tingchao Wu, Rensong Yue.

**Data curation:** Chenyi Xu, Maoyi Yang.

**Formal analysis:** Tao Long, Tingchao Wu.

**Investigation:** Chenyi Xu, Maoyi Yang.

**Methodology:** Tao Long, Tingchao Wu, Rensong Yue.

**Project administration:** Rensong Yue

**Software:** Tao Long, Tingchao Wu.

**Visualization:** Chenyi Xu.

**Writing – original draft:** Tao Long.

**Writing – review & editing:** Rensong Yue.

## References

[1] Krishnasamy S Abell TL Diabetic gastroparesis: principles and current trends in management. Diabetes Ther 2018;9: Suppl 1: 1–42. 10.1007/s13300-018-0454-9 PMC6028327 29934758

[2] Camilleri M Parkman HP Shafi MA American College of Gastroenterology. Clinical guideline: management of gastroparesis. Am J Gastroenterol 2013;108:18–37. 23147521 10.1038/ajg.2012.373 PMC3722580

[3] Kalra S Sharma A Priya G Diabetic Gastroparesis. Diabetes Ther 2018;9:1723–8. 30027528 10.1007/s13300-018-0475-4 PMC6167284

[4] Koch KL Calles-Escandón J Diabetic gastroparesis. Gastroenterol Clin North Am 2015;44:39–57. 25667022 10.1016/j.gtc.2014.11.005

[5] Farmer AD Bruckner-Holt C Schwartz S Diabetic gastroparesis: perspectives from a patient and health care providers. J Patient Cent Res Rev 2019;6:148–57. 31414026 10.17294/2330-0698.1689 PMC6676757

[6] Parkman HP Hallinan EK Hasler WL Nausea and vomiting in gastroparesis: similarities and differences in idiopathic and diabetic gastroparesis. Neurogastroenterol Motil 2016;28:1902–14. 27350152 10.1111/nmo.12893 PMC5125878

[7] Choung RS Locke GR 3rd Schleck CD Risk of gastroparesis in subjects with type 1 and 2 diabetes in the general population. Am J Gastroenterol 2012;107:82–8. 22085818 10.1038/ajg.2011.310 PMC3280088

[8] Kumar M Chapman A Javed S The investigation and treatment of diabetic gastroparesis. Clin Ther 2018;40:850–61. 29748143 10.1016/j.clinthera.2018.04.012

[9] Moshiree B Potter M Talley NJ Epidemiology and pathophysiology of gastroparesis. Gastrointest Endosc Clin N Am 2019;29:1–4. 30396519 10.1016/j.giec.2018.08.010

[10] Aleppo G Calhoun P Foster NC Reported gastroparesis in adults with type 1 diabetes (T1D) from the T1D Exchange clinic registry. J Diabetes Complications 2017;31:1669–73. 28989086 10.1016/j.jdiacomp.2017.08.014 PMC7172031

[11] Ye Y Jiang B Manne S Epidemiology and outcomes of gastroparesis, as documented in general practice records, in the United Kingdom published online ahead of print, Jun 3, 2020. Gut 2020;gutjnl-2020-321277. doi: 10.1136/gutjnl-2020-321277. 10.1136/gutjnl-2020-321277 PMC7948194 32493829

[12] Hirsch W Nee J Ballou S Emergency department burden of gastroparesis in the United States, 2006 to 2013. J Clin Gastroenterol 2019;53:109–13. 29256990 10.1097/MCG.0000000000000972 PMC6005709

[13] Wang S Wang R Zhang Y Therapies for diabetic gastroparesis: a protocol for a systematic review and network meta-analysis. Medicine (Baltimore) 2020;99:e20461. 32481350 10.1097/MD.0000000000020461 PMC7249871

[14] Callaghan BC Little AA Feldman EL Enhanced glucose control for preventing and treating diabetic neuropathy. Cochrane Database Syst Rev 2012;6:Cd007543. 10.1002/14651858.CD007543.pub2 PMC4048127 22696371

[15] McCallum RW Lin Z Forster J Gastric electrical stimulation improves outcomes of patients with gastroparesis for up to 10 years. Clin Gastroenterol Hepatol 2011;9:314–9. e1. 21185396 10.1016/j.cgh.2010.12.013

[16] Heckert J Sankineni A Hughes WB Gastric electric stimulation for refractory gastroparesis: a prospective analysis of 151 patients at a single center. Dig Dis Sci 2016;61:168–75. 26280084 10.1007/s10620-015-3837-z

[17] Wang M Gao YH Xu J Zusanli (ST36) acupoint injection for preventing postoperative ileus: a systematic review and meta-analysis of randomized clinical trials. Complement Ther Med 2015;23:469–83. 26051583 10.1016/j.ctim.2015.03.013 PMC4909358

[18] Wang H Wang L Shi X Electroacupuncture at zusanli prevents severe scalds-induced gut ischemia and paralysis by activating the cholinergic pathway. Evid Based Complement Alternat Med 2015;2015:787393. 26448777 10.1155/2015/787393 PMC4581501

[19] Yang ZK Wu ML Xin JJ Manual acupuncture and laser acupuncture for autonomic regulations in rats: observation on heart rate variability and gastric motility. Evid Based Complement Alternat Med 2013;2013:276320. 24348694 10.1155/2013/276320 PMC3857851

[20] Liu J Huang H Xu X Effects and possible mechanisms of acupuncture at ST36 on upper and lower abdominal symptoms induced by rectal distension in healthy volunteers. Am J Physiol Regul Integr Comp Physiol 2012;303:R209–17. 22592556 10.1152/ajpregu.00301.2010 PMC3404632

[21] Chang SL Lin KJ Lin RT Enhanced insulin sensitivity using electroacupuncture on bilateral Zusanli acupoints (ST 36) in rats. Life Sci 2006;79:967–71. 16762373 10.1016/j.lfs.2006.05.005

[22] Wang LQ Chen Z Zhang K Zusanli (ST36) acupoint injection for diabetic peripheral neuropathy: a systematic review of randomized controlled trials. J Altern Complement Med 2018;24:1138–49. 30431314 10.1089/acm.2018.0053 PMC6987730

[23] Moher D Shamseer L Clarke M Preferred reporting items for systematic review and meta-analysis protocols (PRISMA-P) 2015 statement. Syst Rev 2015;4:1. 25554246 10.1186/2046-4053-4-1 PMC4320440

[24] Guyatt GH Oxman AD Vist GE GRADE: an emerging consensus on rating quality of evidence and strength of recommendations. BMJ 2008;336:924–6. 18436948 10.1136/bmj.39489.470347.AD PMC2335261

